# Live detection and purification of cells based on the expression of a histone chaperone, HIRA, using a binding peptide

**DOI:** 10.1038/srep17218

**Published:** 2015-11-24

**Authors:** K. J. Kochurani, Annie A. Suganya, Madhumathy G. Nair, Jiss Maria Louis, Aditi Majumder, Santhosh Kumar K., Parvin Abraham, Debasree Dutta, Tessy T. Maliekal

**Affiliations:** 1 Cancer Research Program, Rajiv Gandhi Centre for Biotechnology, Thiruvananthapuram, Kerala, India, 695014; 2 Chemical Biology Group, Rajiv Gandhi Centre for Biotechnology, Thiruvananthapuram, Kerala, India, 695014.

## Abstract

Flowcytometry is a reliable method for identification and purification of live cells from a heterogeneous population. Since permeabilized cells cannot be sorted live in a FACS sorter, its application in isolation of functional cells largely depends on antibodies for surface markers. In various fields of biology we find intracellular markers that reveal subpopulations of biological significance. Cell cycle stage specific molecules, metastatic signature molecules, stemness associated proteins etc. are examples of potential markers that could improve the research and therapy enormously. Currently their use is restricted by lack of techniques that allow live detection. Even though a few methods like aptamers, droplet-based microfluidics and smartflares are reported, their application is limited. Here, for the first time we report a simple, cost-effective and efficient method of live sorting of cells based on the expression of an intracellular marker using a fluorophore-tagged binding peptide. The target molecule selected was a histone chaperone, HIRA, the expression of which can predict the fate of differentiating myoblast. Our results confirm that the peptide shows specific interaction with its target; and it can be used to separate cells with differential expression of HIRA. Further, this method offers high purity and viability for the isolated cells.

Identification and isolation of a subpopulation from a heterogeneous cell population has a wide range of biological and medical applications. Currently, the cell detection and isolation is mainly dependent on antibodies for a particular protein. Even though flowcytometry is a powerful tool that helps to purify a cell type based on markers, its application is limited to cell surface proteins, since the detection is mainly based on antibodies. Live sorting of cells using antibody for internal markers is not possible because permeabilization is required for antibody to detect internal molecules. An alternative approach used is the use of a fluorescent substrate for an enzyme, like aldehyde dehydrogenase^1^, which has given a new dimension to stem cell research and therapy. Use of aptamers, where a library of aptamers has to be tested to select a suitable aptamer for each cell type, is also an attractive strategy in specific cell culture models^2^. A wide use of that strategy is not applicable to cell types where there is heterogeneity. Another attractive strategy reported for sorting based on and limited to secreted molecules is droplet-based microfluidics^3^. Recently cell detection based on RNA has evolved, and multiplexed nanoflares^4^ are reported for detection, and molecular beacons are reported for purification of cells^5^, where the introduction of the beacons depends on microinjection, streptolysin O or electroporation. Recently, a combination of protein cell surface markers and smartflares,which are RNA binding nucleotides linked to gold nanoparticle, are reported to be useful in live isolation of prostate cancer cells^6^. These smartflares are RNA-binding nucleotides, and their use is limited to mRNA-based markers. In many instances, the expression of protein and its mRNA do not have one to one correlation due to posttranscriptional regulation^7^,^8^. Thus there is inadequacy in the methods available in fractionating cells based on their differential expression of internal molecules like transcription factors, nuclear chaperones and other signaling intermediates that reflects functional heterogeneity.

From the time of identification of cyclins, it has been established that several proteins oscillate in a cell cycle-dependent manner. Introduction of FUCCI reporter system based on this information enabled identification and isolation of each cell cycle stage, and made it possible to chase the cells at different stages^9^. Exploiting this system in developmental biology, it has been shown that heterogeneity in pluripotent cells mainly rely on cell cycle stages^10^. Embryonic stem (ES) cells in early G1 phase differentiate to endoderm and mesoderm, and late G1 cells differentiate to neuroectoderm, while G2/S/M cells do not respond to differentiation signals^11^. This information can drastically improve stem cell therapy provided we have a reagent to isolate live cells at different cell cycle stages. Since FUCCI reporter system does not work on primary stem cells isolated from a patient, a method to isolate live cells based on cell cycle stage specific markers is warranted.

Cancer cells grow in the primary site and a subset of cancer cells acquire invasive and metastatic property to spread to secondary sites. Immense efforts were taken to understand the molecular players involved in metastasis. With microarray analysis and immunohistochemical analysis of a cohort of patient samples, several sets of genes have been identified that regulate metastasis and specify the site of metastasis^12^,^13^,^14^,^15^. The validations of these markers are done in cell lines by over-expressing or knocking out these genes. ID1 expression is one of the parameters that dictate metastasis of breast cancer cells to lung^14^. Validation of metastatic potential of a subpopulation of tumor cells over-expressing ID1 in a primary tumor sample is not possible due to lack of a method to isolate live cells based on ID1 expression. Taken together, in the present scenario a simple and efficient method to detect live cells based on internal protein markers can offer an improvement in a wide area of research and therapy.

Differentiation of ES cells from a pluripotent to a committed state involves global changes in genome expression patterns, where histones and histone binding proteins play a major role^16^. A pluripotent mesenchymal stem cell can differentiate to osteoblasts, chondrocytes, adipocytes and myoblasts. C2C12myoblast cell line is a system used to study differentiation, either to myotubes or to osteoblasts depending on the culture condition. It has been reported that histones and histone chaperones (like CAF1, Asf1 and HIRA) are important factors regulating skeletal muscle formation^17^. Interestingly, it has been shown that the expression of HIRA and Asf1a is unchanged during myogenic differentiation, while their expression considerably decreases during osteogenic conversion^18^. This information could be used to identify committed cell types to muscle cell or bone cell during differentiation, provided we have a means to isolate cells based on the expression of these nuclear chaperones. Since HIRA is an important molecule that can predict the fate of myoblasts, we selected this molecule for designing probes to detect and isolate live cells based on its expression.

Since the protein-protein interaction depends on the affinity of small peptide regions within the protein molecules, synthetic peptides that have affinities to specific targets emerged as a new tool for protein targeting^19^. Now the binding peptides are widely used in imaging and therapy^20^,^21^. The major advantages of peptides are that it is easy to synthesize and modify them, and are relatively nontoxic and cell penetrating depending upon the composition of amino acids. Cell penetrating peptides (CPP) are small peptides less than 35 amino acids, possessing limited cytotoxicity; ability to facilitate receptor-independent transport across cell membranes; and ability to cross the cell plasma membrane in both energy-independent processes and endocytosis. A large number of CPP has highly positive net charges that originate from the basic short strands of arginines and lysines. With its increasing demand in drug delivery and imaging, considerable efforts were put in for the design and synthesis of CPPs^22^,^23^. The types of CPPs and their mechanism of action are reviewed recently^23^. Here, we designed a binding peptide for HIRA that could specifically bind to the target molecule, and with a tagged fluorophore, it can be used to detect and isolate live cells.

## Results and Discussion

### Design and synthesis of the peptide

**Figure 1:**
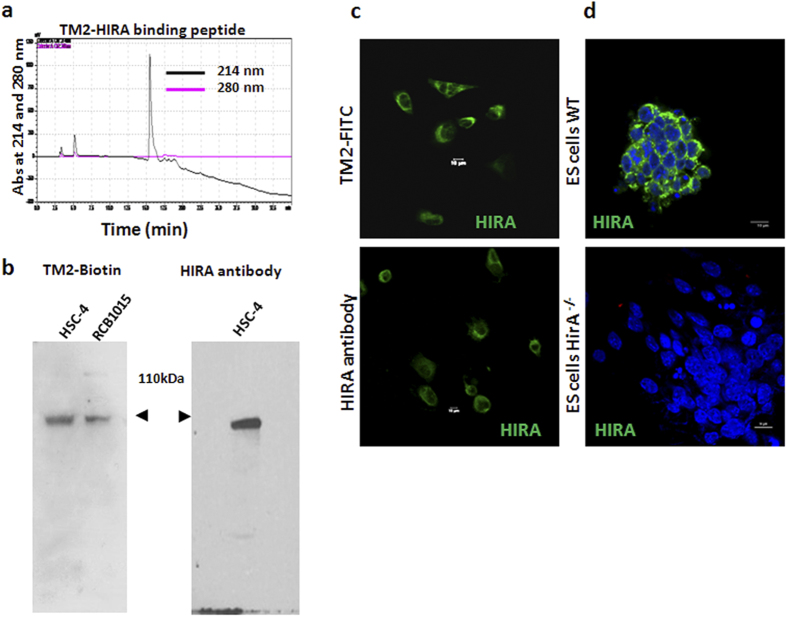
Purification and confirmation of specificity of TM2. (**a**) The RP-HPLC profile of the synthesized peptide. TM2 was eluted at 15.5 minutes. (**b**) The western blot analysis for testing the specificity of the peptide-targeting using biotin-tagged peptide in oral cancer cell lines. The right panel shows the western blot using HIRA antibody. (**c**) Immunofluorescence detection of HIRA in fixed HSC-4 cells using TM2-FITC or HIRA antibody. (**d**) Immunofluorescence of fixed ES cells using TM2-FITC. Hoechst 33342 dye was used to visualize the nucleus. The scale bar represents 10 μm.

For designing binding peptides, the proteins that interact with HIRA were searched using Human Protein Reference Database (http://www.hprd.org/). Out of the six molecules, H2B, which binds to minimum partners was selected. From the literature the binding sequence of H2B that is essential for HIRA-H2B interaction was selected, which does not overlap with its interaction with FACT, another histone chaperone^24^,^25^. The success of the design of specific interacting peptide is dependent on the selection of the interacting molecule and the region that interacts with the target molecules. Even though the success rate is very high in this method, this approach is limited to the molecules where the interacting regions are reported. For other molecules bioinformatics tools like PeptideMine (http://caps.ncbs.res.in/peptidemine/example_runs.html) can be used to design binding peptides. The sequence of the region selected was used to design peptides using PeptGen peptide generator (http://www.hiv.lanl.gov/content/sequence/PEPTGEN/peptgen.html). Since it is known that peptides having net positive charges show more cell permeability, we selected PKKGSKKAVTKAQKKDGA for synthesis of TM2 peptide. Fmoc solid phase peptide synthesis protocol using polymer support was used to synthesize the peptide. After the synthesis, the peptide was cleaved from the support and its purity was analyzed by HPLC C18 reverse-phase column (Fig. 1a).

### Confirmation of the specificity of TM2

One of the problems that could emerge in a peptide-based detection is its specificity. To confirm whether the peptide detects correct molecule, we performed a western blot using biotin-tagged peptide, a novel approach for showing specificity. TM2 was tagged with biotin and purified by size exclusion chromatography, and was used instead of primary antibody in western blot. The blot was developed with streptavidin-HRP and ECL to get a single band at 110 kDa, as observed in antibody-based detection (Fig. 1b). The peptide was tagged to FITC and was used for immunofluorescence in comparison with HIRA antibody. The staining pattern in fixed and permeabilized oral cancer cell line HSC-4 was similar in both TM2-FITC and HIRA antibody (Fig. 1c), confirming the specificity. To rule out nonspecific binding of peptide we selected HIRA-knock out cells. So, we analyzed the specificity using mouse W9.5 (wild type) and *Hira*^−/−^ W9.5 ES cells, reported earlier^26^,^27^. Since we designed peptides using human sequence, and the cell line is of mouse origin we compared the sequence similarity of binding region of both HIRA and H2B across the two species. The binding region of H2B encompassing the designed peptide has 93.2% sequence similarity with mouse sequence , while the HIRA region that binds to H2B shows 92.8% sequence similarity when compared with CLUSTALW (http://www.ebi.ac.uk/Tools/msa/clustalw2/). So we anticipated that the peptide will recognize mouse HIRA as well. Immunofluorescent staining of fixed and permeabilized ES cells with TM2-FITC showed that TM2-FITC binds only to HIRA, as evidenced by absence of staining in HIRA^−/−^ ES cells (Fig. 1d).

### Evaluation of cell permeability of TM2-FITC

**Figure 2:**
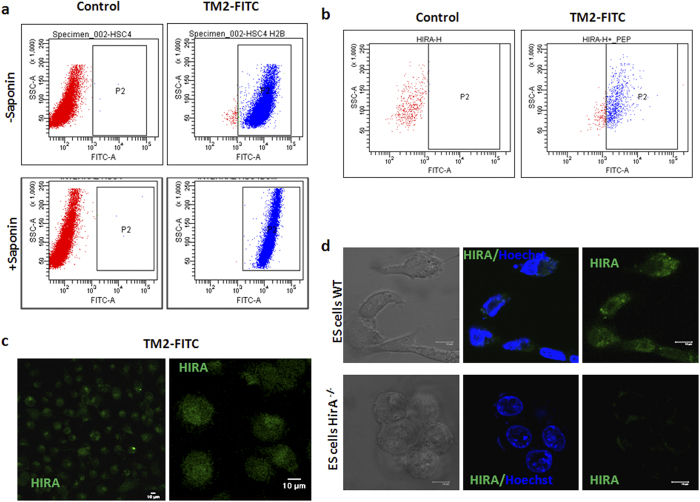
TM2 is cell permeable and shows specificity in target detection. (**a**) The FACS profile of fixed HSC-4 cells stained with TM2-FITC. (**b**) Live cells were stained with the TM2-FITC as described under methods and analyzed in FACS Aria II. (**c**) HSC-4 cells were grown in confocal dishes and incubated with TM2-FITC diluted in PBS for 1 h. Prior to imaging the cells were briefly washed and imaged (**d**) ES cells were grown in confocal dishes and stained using TM2-FITC. Hoechst 33342 dye was used to visualize the nucleus. The scale bar represents 10 μm.

TM2-FITC was used for FACS analyses with and without permeabilization of fixed HSC-4 cells using saponin. The staining pattern confirms that the peptide is internalized without permeabilization, and can be used to isolate cells by FACS (Fig. 2a). To check whether it can be used for live sorting, live HSC-4 cells were stained with the peptide and analyzed by FACS. The results show that 85% of cells are positive for HIRA (Fig. 2b). An important observation was the loss of the signal when washed with peptide free solution, possibly because the equilibrium of uptake and export of the peptide is lost. The live uptake of TM2-FITC by HSC-4 monolayer cells was visualized under microscope immediately after washing off the staining solution (Fig. 2c). The live uptake pattern of ES confirmed the specificity of live uptake, since the uptake was limited to the wild type ES cells (Fig. 2d).

### Live sorting using TM2-FITC

**Figure 3:**
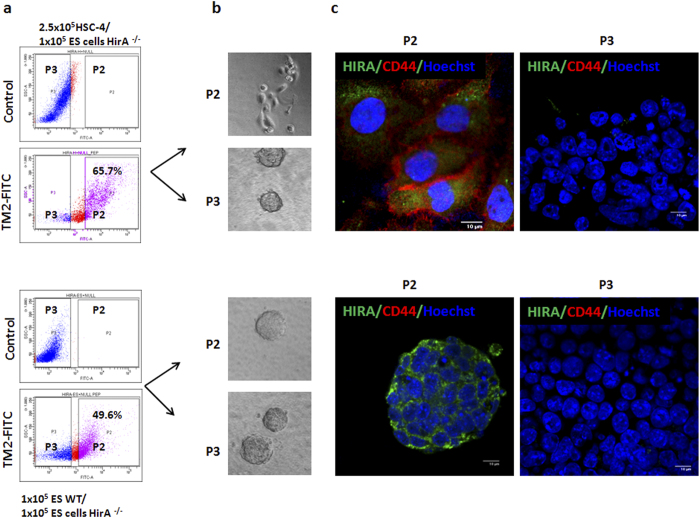
TM2-FITC live sorting. (**a**) The FACS profile of a mixture of live HSC-4 cells and ES cells stained with TM2-FITC. The two populations in P2 and P3 gate were sorted. (**b**) Sorted cells were grown in 8-well chamber slide for 5 days, and DIC images were taken (**c**) the 5-day old sorted cells were stained with indicated antibodies after fixation with 4% PFA. The scale bar represents 10 μm.

**Figure 4:**
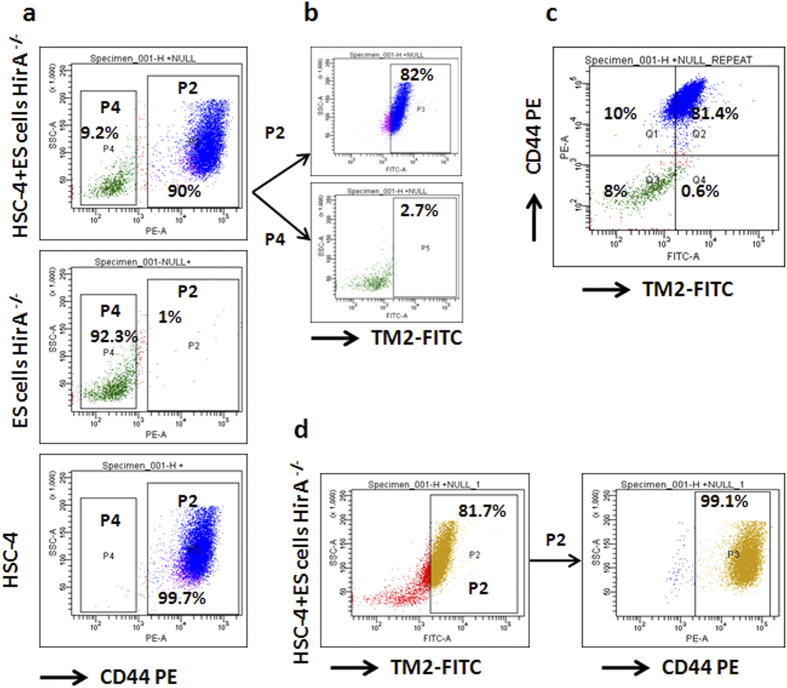
Specificity of TM2-FITC live sorting. (**a**) The FACS profiles of live HSC-4 cells and ES cells alone or in a mixture stained with TM2-FITC and CD44-PE. The two populations in P2 and P4 gate were selected for CD44 positivity. (**b**) Sorted cells were further analyzed for HIRA positivity (**c**) The dual stained cells were separated for both FITC and PE. The positivity of different populations is marked. (**d**) The mixture of two cell types were separated for HIRA positivity and the P2 population was further analyzed for CD44 positivity.

Since TM2-FITC binds to its target in live cells without any permeabilizing agents, we sought the possibility of its use in live sorting. We tried to separate HIRA expressing cells from HIRA deficient cells using FACS. We took oral cancer cell line HSC-4, which expresses HIRA, and mixed with *Hira*^−/−^ ES cells in 2.5:1 ratio. We observed 65.7% HIRA positive cells against the expected 71.4% (Fig. 3a). These cells were sorted out as positive and negative populations with gates P2 and P3 and were allowed to grow for 5 days in 8-well chamber slide. The DIC images confirm that the positive population was HSC-4 keratinocytes and the negative population was ES cells (Fig. 3b). Again wild type ES cells and *Hira*^−/−^ ES cells were mixed in 1:1 ratio and sorted as mentioned before. The observed ratio of positivity was 49.6% against expected ratio of 50% (Fig. 3a). The ES cells were viable and formed colonies in 5 days as shown in Fig. 3b. These cells grown in slide chambers were stained with antibodies for HIRA and an epithelial marker, CD44. The results showed that the P2 population representing HSC-4 cells were all positive for HIRA and CD44, while the P3 population showed negativity for both molecules (Fig. 3c). Likewise, in the separation of wild type and *Hira*^−/−^ ES cells the fractionation was successful as the colonies of P2 population showed positivity for HIRA, and negative population did not show any staining. The P3 population in the mixture of ES cells and HSC-4 cells, however, showed  <1% of contamination from CD44^+^HIRA^+^cells. The cross contamination in P3 population for the mixture of null and wild type ES cells was <4%. Since 5 days of growth in ES culture medium might give a selection advantage for ES cells over the contaminating keratinocytes, another FACS was performed to check the false positivity and negativity. HSC-4 cells and *Hira*^−/−^ ES cells were stained alone or in mixture with the TM2-FITC and CD44-PE and analyzed by FACS. All the HSC-4 cells were positive for CD44, while 1% of *Hira*^−/−^ ES cells showed a false positivity for CD44 (Fig. 4a). In a mixture of both the cell types, the cells that showed CD44 positivity (P2) or negativity (P4) were analyzed for HIRA positivity. Majority of CD44^+^ population (82%) showed HIRA positivity while a very small fraction of *Hira*^−/−^ ES cells (2.7%) showed false positivity (Fig. 4b). Figure 4c shows how the two populations separate out in a dual staining. The false positivity that we observed in P4 population could be overcome by a stringent gating as we have done in Fig 3. To further ascertain the false positivity in sorting based on TM2-FITC, the mixture of two cell types were separated for HIRA a positivity as shown in Fig. 4d. The positive population was analyzed for possible cross contamination for ES cells as shown by CD44 negativity. The false positive ratio was 0.9% as the rest of the cells were CD44^+^ (Fig. 4d) comparable to the 0.6% shown in Fig. 4c. Taken together, our results show that designing a suitable binding peptide and tagging it with a fluorophore is a powerful tool to detect and purify live cells based on internal markers. Comparing the cost of peptide synthesis with antibody development, this method is more cost effective than conventional antibodies. In future, the strategy can be improved by modifications that increases the retention time of the peptide, to ensure more purity. We argue that this approach will have immense use in cell biology, developmental biology and clinical applications.

## Methods

### Synthesis of peptide

Stepwise manual solid phase peptide synthesis technique using CLEAR amide resin as the solid support and following the 9-fluorenylmethoxy carbonyl (Fmoc) chemistry was used to synthesize C-terminally amidated peptide^28^. After the synthesis, a cleavage cocktail TFA/EDT/TIS/water (95:1.5:1.5:2 (v/v)) was added to cleave peptide amide from the resin. The crude peptide amide was precipitated by ice cold ether. After lyophilisation it was dissolved in solvent A (100% water, T0.1% TFA) and purified by RP-HPLC using C18 column. The peptide was eluted out of the column by slowly increasing the acetonitrile concentration to 70% in solvent A over a period of 30 min. Homogeneous purification and mass accuracy were confirmed by analytical RP-HPLC. Labeling was achieved by addition of 25 μl of FITC (1 mg/ml) in DMSO to 1 ml of a peptide solution (1 mM) in water, adjusted to pH 9.7 with Na_2_CO_3._ After overnight incubation at 4 °C in the dark, residual FITC was inactivated by incubation with 50 μl of 1 M NH_4_Cl for 2 h. For the biotinylation of the peptide, the peptide and biotin were taken in 1:5 ratio, and the reaction was done using a Biotinylation kit (GeNei, India) following the manufacturer’s protocol. The peptide conjugates were stored in aliquots at −20 °C until use.

### Western Blotting

Western blotting was performed as described before^29^. Briefly, cell lysates were prepared in RIPA lysis buffer and the bands were resolved by 10% SDS-PAGE, and transferred to nitrocellulose membrane for immunoblot analysis. Biotin-tagged peptides (2 μM) were added, and the bound peptide was detected using streptavidin-HRP. Alternatively, primary and corresponding secondary antibody linked to HRP were used. The blot was developed using GE Healthcare ECL Plus western blotting developing system and imaging was done using Versa Doc 4000MP (BioRad).

### Maintenance of ES culture

The wild type and *Hira*^−/−^ ES cells were cultured in ES-IMDM medium with LIF (10^6^ units/ml) for 3 days^26^. For the initial passages, cells were grown on mitomycin inactivated mouse embryonic fibroblasts as feeder layer while for the experiments the cells were cultured on gelatin coated plates.

### Immunofluorescence

Monolayer cells seeded in micro slide chamber were fixed using 4% paraformadehyde (PFA), permeabilized with 0.3% Triton X-100/PBS (PBST) and blocked with 2% serum as described previously^30^. Primary antibody or peptides tagged with FITC were added and incubated at 4°C overnight. Secondary antibody was added and incubated for one hour for antibody added cells. Hoechst 33342 was used to stain the nuclei, and antifade was used to mount. Images were acquired using NikonA1R LSCM confocal microscope.

### FACS

FACS analysis was performed as reported previously^30^. Monolayer cells were trypsinized and fixed using 4% PFA, and peptide labeled with FITC (10 μM) was added to the cells in presence or absence of 0.3% saponin in a buffer containing 1%FBS, and incubated for one hour at room temperature. For live sorting, the cells were resuspended in 75 μl of PBS containing the peptide for one hour. Cells were diluted with 200 μl of PBS and sorted immediately using FACS Aria II.

## Additional Information

**How to cite this article**: Kochurani, K. J. *et al.* Live detection and purification of cells based on the expression of a histone chaperone, HIRA, using a binding peptide. *Sci. Rep.* **5**, 17218; doi: 10.1038/srep17218 (2015).
